# Laparoscopic Mesh Repair of a Strangulated Right Obturator Hernia With Intraoperative Indocyanine Green Assessment of Bowel Viability: A Case Report

**DOI:** 10.7759/cureus.96211

**Published:** 2025-11-06

**Authors:** Roberto Del Giudice, Gianluca Mazzoni, Chiara Tranfaglia, Annamaria Bellotti, Pierandrea Zofrea

**Affiliations:** 1 General Surgery, Azienda Sanitaria Locale (ASL) Roma 3, Rome, ITA; 2 Surgery, Sapienza Università di Roma, Rome, ITA

**Keywords:** bowel obstruction, emergency laparoscopy, indocyanine green (icg), little old lady's hernia, obturator hernias

## Abstract

Obturator hernia is a rare condition. Due to its deep pelvic location, surgical management is often challenging. We present the case of an elderly woman admitted with abdominal pain, vomiting, and no passage of stool or flatus for five days. Computed tomography revealed a right obturator hernia with an incarcerated ileal loop. A laparoscopic transabdominal preperitoneal (TAPP) repair was performed. After peritoneal incision and dissection of the Retzius and Bogros spaces, the incarcerated bowel loop was reduced. A self-gripping polyester mesh was placed to cover the myopectineal orifice and obturator foramen, and the peritoneal flap was closed with an absorbable barbed suture. Intestinal perfusion was assessed intraoperatively using indocyanine green (ICG) fluorescence, which demonstrated a 3 cm infarcted segment requiring resection and hand-sewn, two-layer isoperistaltic anastomosis through a mini-umbilical incision. The postoperative course was uneventful with discharge on day five. This case highlights the feasibility and safety of the laparoscopic approach for strangulated obturator hernia, even in frail patients, and underscores the usefulness of ICG fluorescence in assessing bowel viability and avoiding unnecessary resections.

## Introduction

Obturator hernia, first described by Pierre Roland Arnaud de Ronsil in 1724, is a rare condition accounting for 0.07%-1% of all hernias and approximately 0.4% of mechanical intestinal obstructions [1,2]. It occurs when intra-abdominal contents protrude through the obturator canal, a musculoaponeurotic defect approximately 0.2-0.5 cm in diameter and 2-3 cm in length that contains the obturator nerve, artery, and vein, located in the cranial portion of the obturator foramen, bounded superolaterally by the superior pubic ramus and inferiomedially by the obturator membrane and the obturator muscles [3,4]. The main predisposing factors include advanced age, female sex, low body mass index, multiparity, and pelvic floor weakness. For these reasons, obturator hernia is often referred to as the “little-old-lady’s hernia” [4-6]. Due to the deep anatomic location of the obturator canal, obturator hernia is rarely detectable on physical examination. In approximately 60% of cases-when both emergent and non-emergent presentations are considered-the diagnosis is established intraoperatively during exploratory laparotomy or laparoscopy [7]. Given its rarity and non-specific clinical presentation, early recognition remains difficult. The increasing adoption of minimally invasive approaches has made laparoscopy a valuable diagnostic and therapeutic option, particularly in emergency settings. Moreover, the intraoperative use of indocyanine green (ICG) fluorescence imaging has emerged as a useful adjunct for real-time assessment of bowel perfusion, allowing more accurate evaluation of intestinal viability. This case report describes a patient with a strangulated right obturator hernia successfully treated with a laparoscopic transabdominal preperitoneal (TAPP) approach and intraoperative ICG-assisted evaluation of bowel vitality.

## Case presentation

An elderly woman presented to the emergency department with a five-day history of abdominal pain, vomiting, and absence of passage of stool and flatus. The patient was afebrile and hemodynamically stable on admission. Her past medical history included atrial fibrillation on oral anticoagulation and Parkinson’s disease, with no previous abdominal surgeries. On examination, she appeared asthenic and sarcopenic. Abdominal distension and diffuse tenderness were noted without signs of peritonitis. No inguinal or femoral hernias were palpable. A nasogastric tube was inserted, draining approximately 800 mL of enteric material. The patient presented with leukocytosis, elevated inflammatory markers (C-reactive protein (CRP)), electrolyte imbalances, and a borderline elevated lactate level, suggestive of early tissue hypoperfusion (Table 1).

**Table 1:** Admission laboratory results This table summarizes the patient’s admission laboratory findings. Reference ranges are based on standard hospital values. Abnormal values suggest leukocytosis, systemic inflammation, mild hypernatremia, and a slightly elevated lactate level, possibly indicating early tissue hypoperfusion.

Test	Patient result	Reference range	Units
White blood cells (WBC)	17,000	4,000–10,000	cells/μL
C-reactive protein (CRP)	18.2	<0.5	mg/dL
Potassium (K⁺)	3.1	3.5–5.0	mEq/L
Sodium (Na⁺)	149	135–145	mEq/L
Lactate	2.2	0.5–2.0	mmol/L

**Figure 1:**
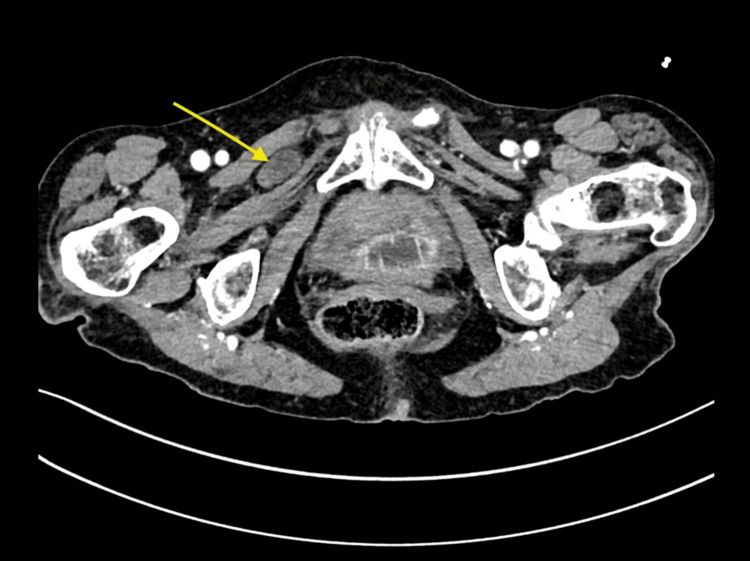
Axial CT scan showing right obturator hernia with incarcerated ileal loop This axial slice from a contrast-enhanced abdominal CT demonstrates a loop of ileum (arrow) herniating through the right obturator canal. The ileal loop is located between the pectineus and the obturator externus muscle. CT: computed tomography

**Figure 2:**
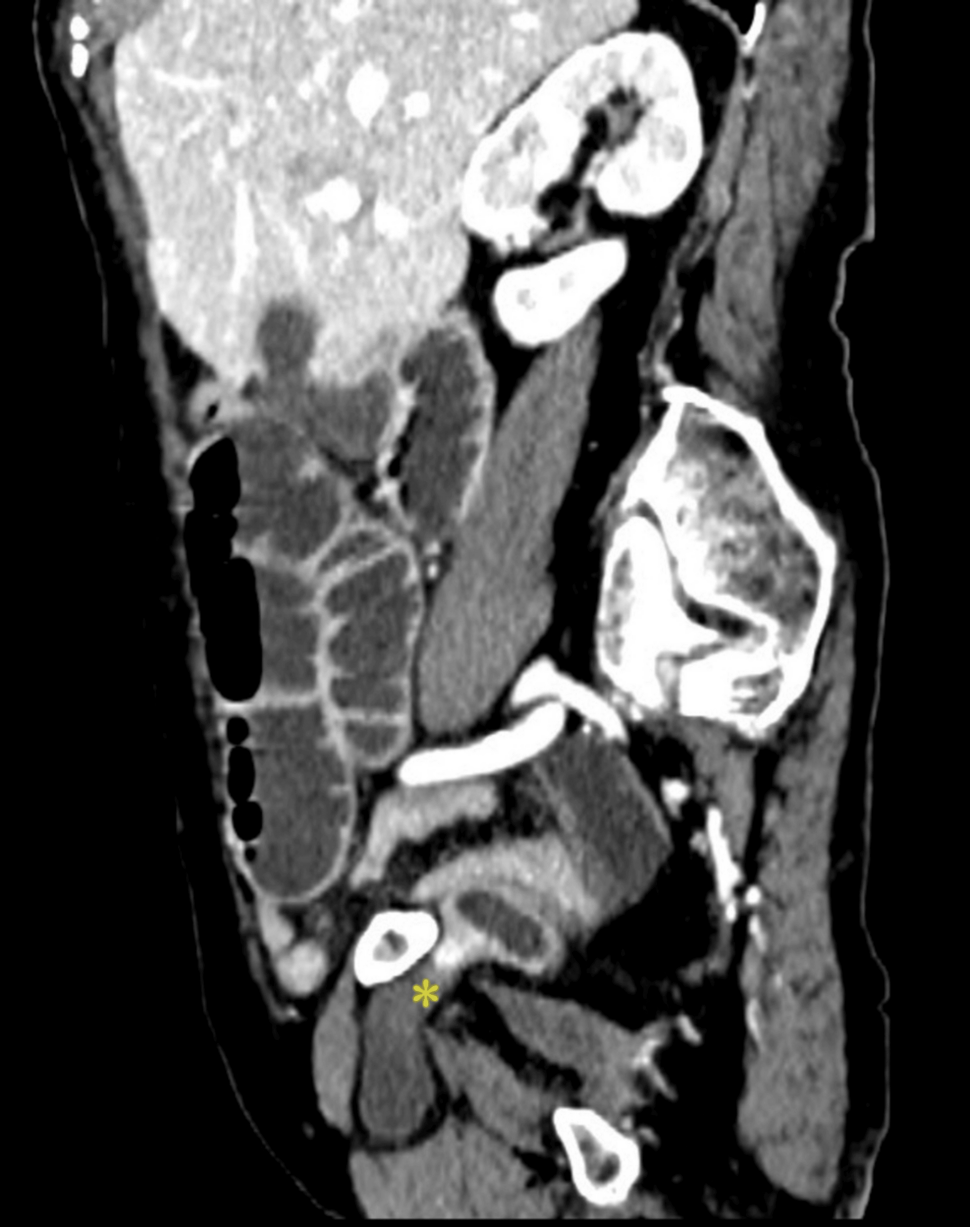
Sagittal CT scan demonstrating the ileal loop protruding through the obturator canal (asterisk) This sagittal CT image shows the herniated ileal loop (asterisk) passing through the right obturator canal. The anatomical boundaries of the canal are clearly visible, with the superior pubic ramus forming the roof, and the obturator internus (deep) and obturator externus (superficial) muscles forming the inferior and posterior margins. The herniated loop is contained in a space bordered anteriorly by the pectineus muscle, posteriorly by the obturator externus, and inferiorly by the adductor muscles. Dilated intestinal loops are also visible, consistent with mechanical bowel obstruction. CT: computed tomography

Because the clinical findings suggested mechanical small-bowel obstruction, an abdominal computed tomography (CT scan) with contrast was performed. The scan demonstrated a right-sided obturator hernia containing an incarcerated ileal loop (Figure 1: axial CT view; Figure 2: sagittal CT view). Given the diagnosis, an emergency laparoscopic exploration was undertaken using an umbilical port for initial access. Intraoperatively, the obstruction was confirmed to be caused by an ileal loop incarcerated within the right obturator canal (Video 1). Two additional 5 mm trocars were placed in the right and left flanks, following the usual setup for laparoscopic inguinal hernia repair.

**Video 1:** Laparoscopic reduction of the strangulated obturator hernia and intraoperative indocyanine green fluorescence assessment of bowel perfusion The video presents the clinical case, including a brief introduction and preoperative CT images and clips. Intraoperative footage demonstrates each key step of the procedure. After an initial unsuccessful attempt to reduce the herniated loop, a right peritoneal flap is incised. The dissection proceeds through the Bogros and Retzius spaces to expose the obturator foramen, which still contains the incarcerated ileal loop. Division of the medial constricting ring allows for reduction of the loop and subsequently the hernia sac. A self-gripping polyester mesh is then placed to reinforce the area. Finally, indocyanine green (ICG) fluorescence is used to assess the viability of the ileal loop. CT: computed tomography

**Figure 3:**
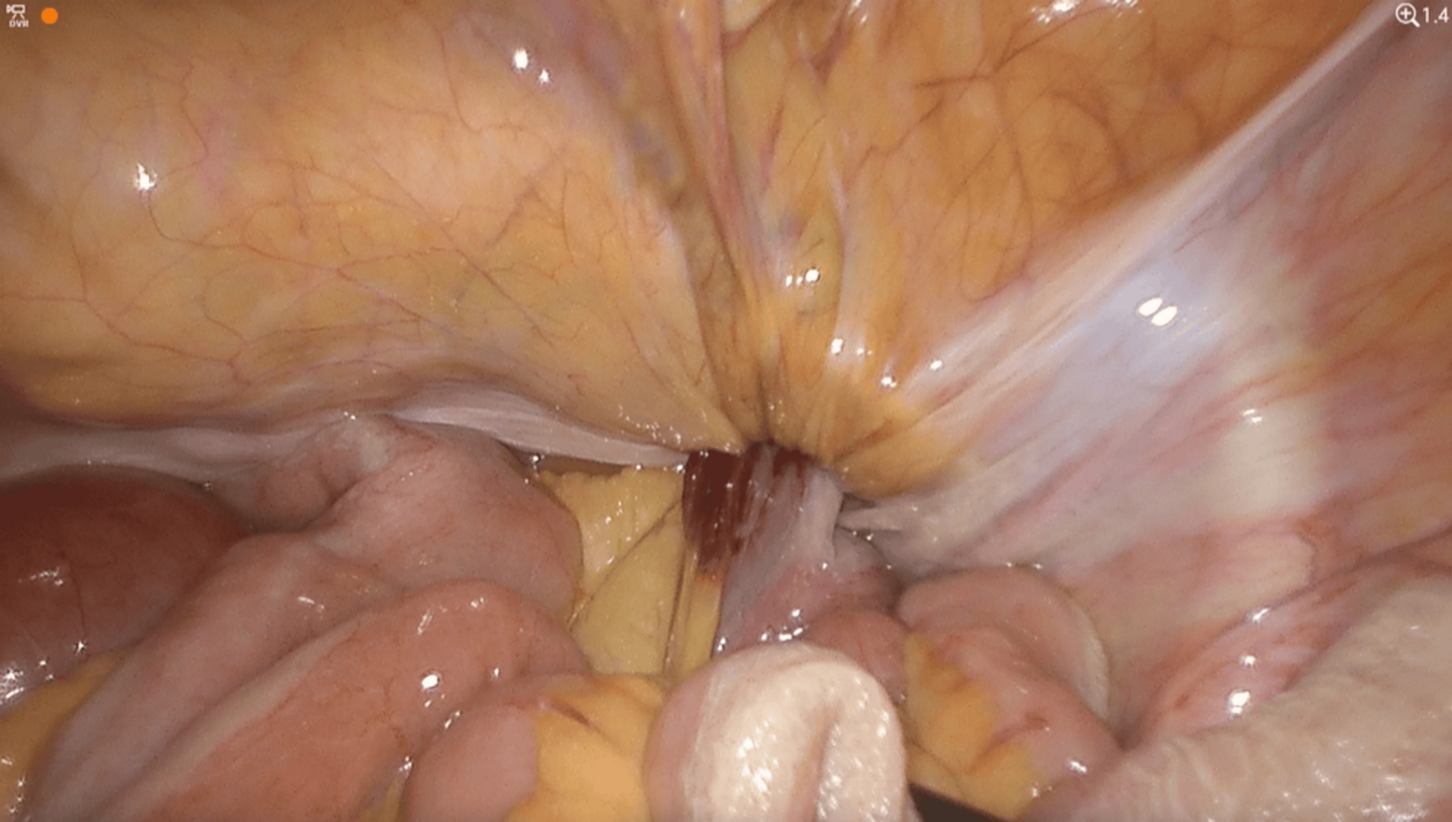
Intraoperative view showing the ischemic ileal loop incarcerated in the obturator canal This intraoperative laparoscopic image shows an ileal loop herniated through the right obturator canal. The loop appears violaceous, indicating early ischemia. The surgeon’s grasper is gently pulling the mesentery of the distal (non-dilated) portion of the loop to avoid traction injury. Despite these efforts, the loop could not be reduced through traction alone. Therefore, we proceeded with a preperitoneal dissection to access the obturator canal, followed by careful division of the constricting fibrous ring.

**Figure 4:**
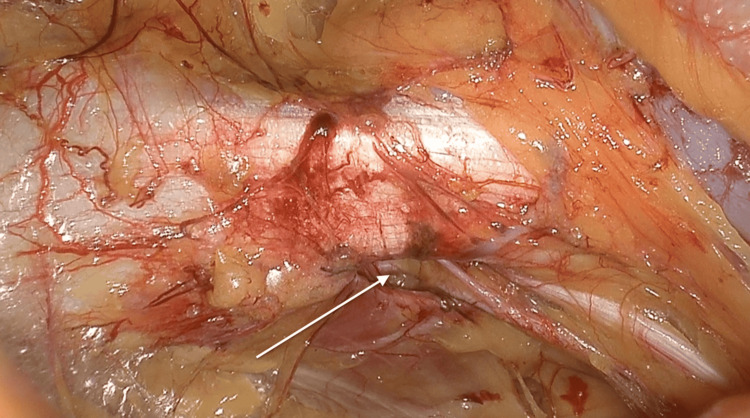
Dissection and exposure of the right obturator canal (arrow) prior to mesh placement This image shows a clear preperitoneal laparoscopic dissection of the right obturator canal, indicated by the arrow. The superior margin is defined by a groove on the inferior pubic ramus, while the inferior border is formed by the obturator internus muscle. The obturator artery, vein, and nerve are also visible as they pass through the canal, demonstrating the critical neurovascular structures at risk during hernia reduction.

**Figure 5:**
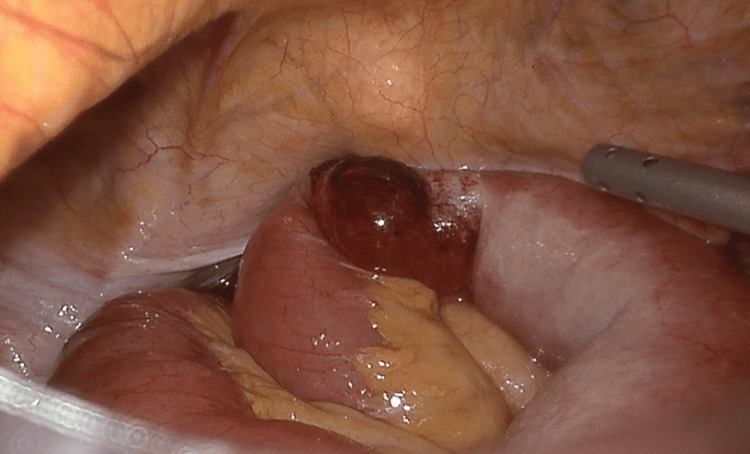
Reduced ileal loop After laparoscopic reduction of the incarcerated bowel loop, an approximately 2 cm segment of ileum appears violaceous, raising suspicion for ischemic injury. Given the patient’s advanced age and the uncertainty regarding bowel viability, we opted to assess perfusion using indocyanine green (ICG) fluorescence to avoid an unnecessary resection.

**Figure 6:**
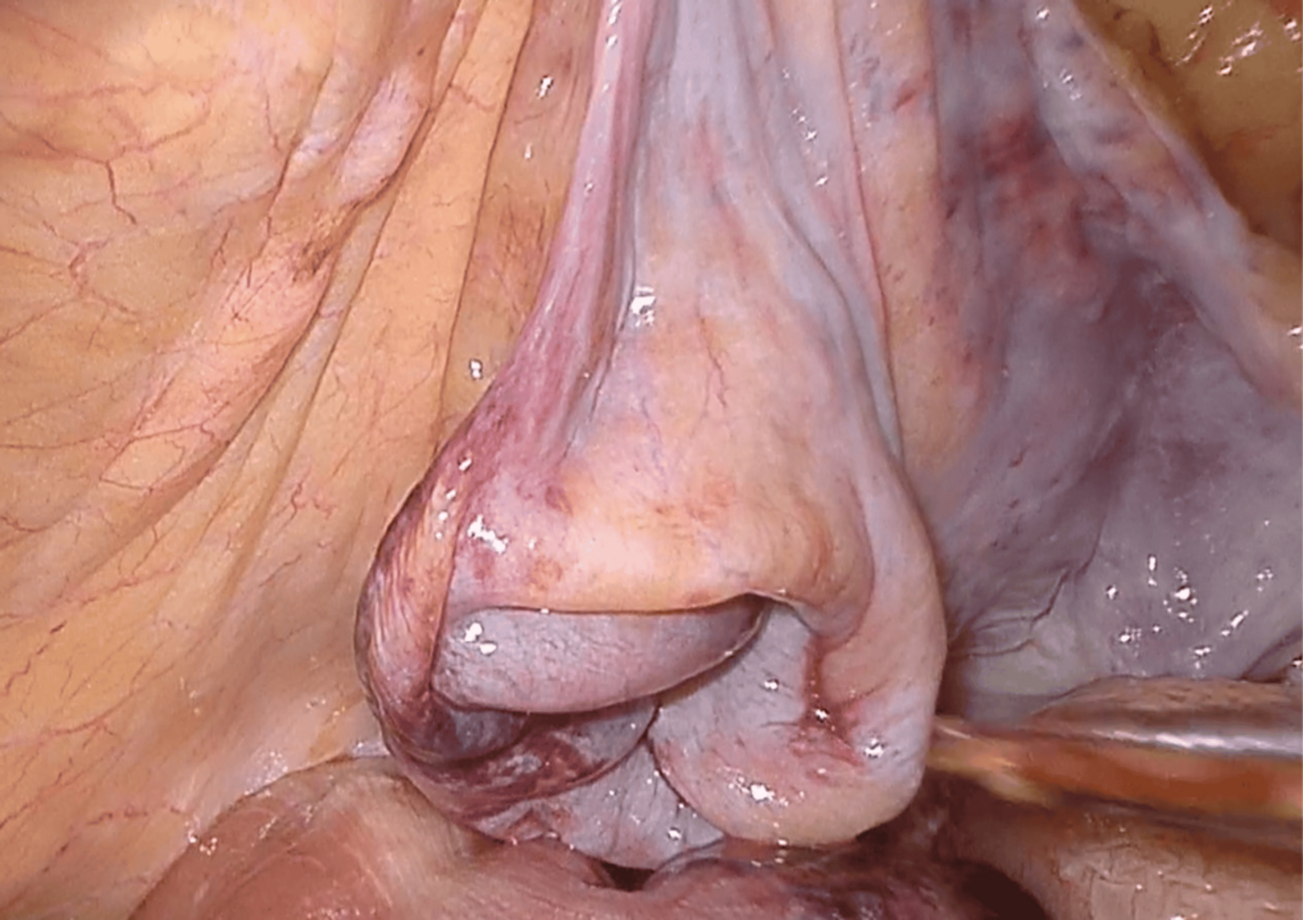
Hernia sac after reduction showing ischemic changes After reducing the incarcerated ileal loop, the hernia sac was also identified and reduced. It appears centrally in the image, contiguous with the inferior peritoneal flap, and shows focal areas of ischemic discoloration. Despite these findings, no resection was deemed necessary. The sac was ultimately incorporated into the re-peritonealization suture of the flap.

**Figure 7:**
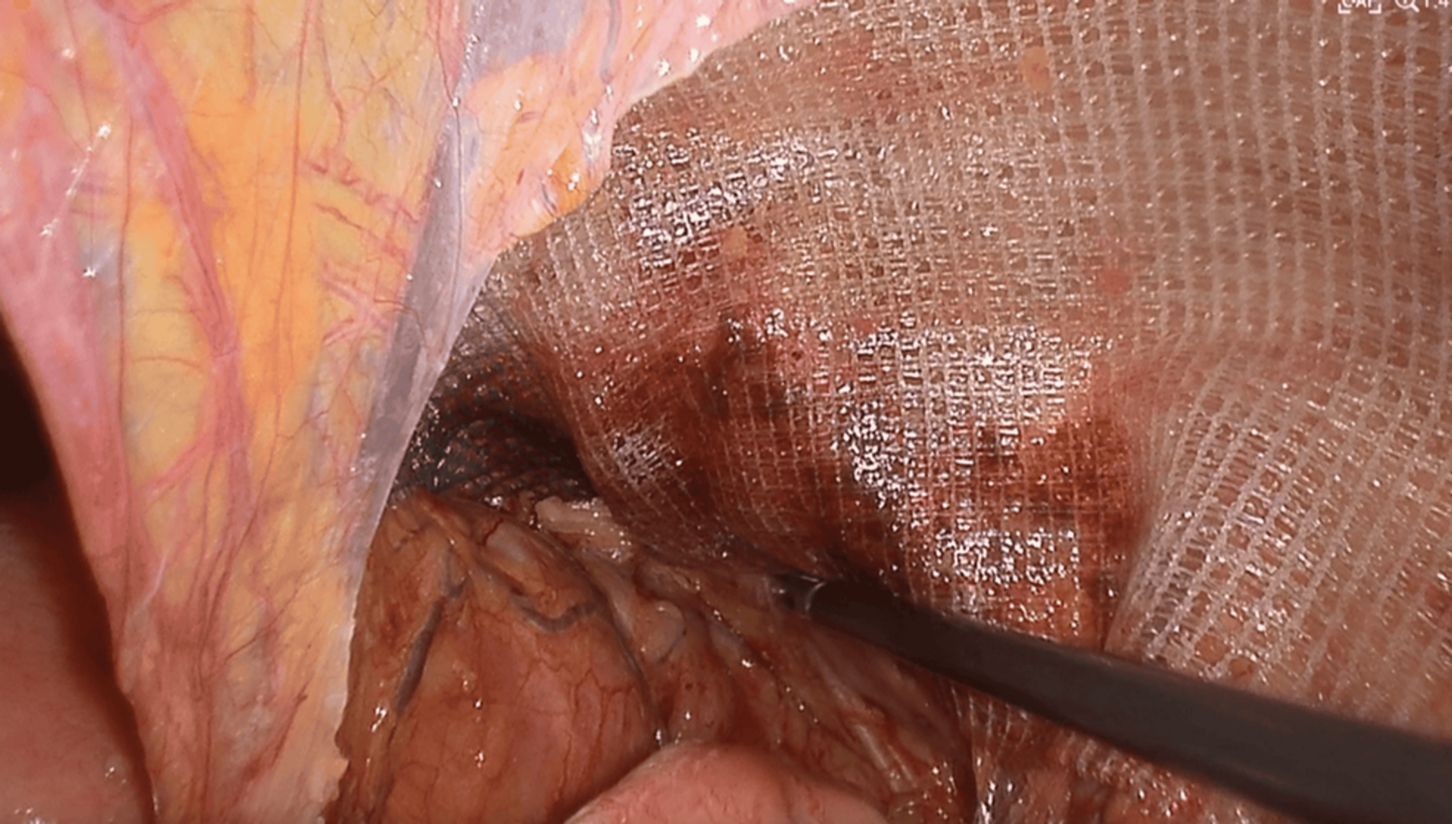
Self-gripping polyester mesh covering the myopectineal orifice of Fruchaud and the obturator foramen The hernia was repaired using a self-gripping polyester mesh measuring 10 cm × 15 cm. The prosthesis is shown well-positioned, fully covering both the obturator foramen and the myopectineal orifice of Fruchaud. This placement addresses the obturator hernia while simultaneously reinforcing the inguinal and femoral regions to prevent future herniation.

Initial attempts to manually reduce the incarcerated loop were unsuccessful (Figure 3), so the parietal peritoneum was incised from the anterior superior iliac spine to the umbilical ligament, similar to the TAPP approach. Dissection was extended within the Bogros and Retzius spaces to expose the right obturator canal (Figure 4). After careful division of adhesions in the medial portion of the canal, the incarcerated ileal loop was successfully reduced (Figure 5). The bowel appeared ischemic, and the hernia sac also showed signs of congestion (Figure 6). The round ligament was divided, and the preperitoneal plane was further developed to accommodate a self-gripping polyester mesh covering the myopectineal orifice of Fruchaud and the obturator foramen (Figure 7). The peritoneal flap was closed with an absorbable barbed suture.

**Figure 8:**
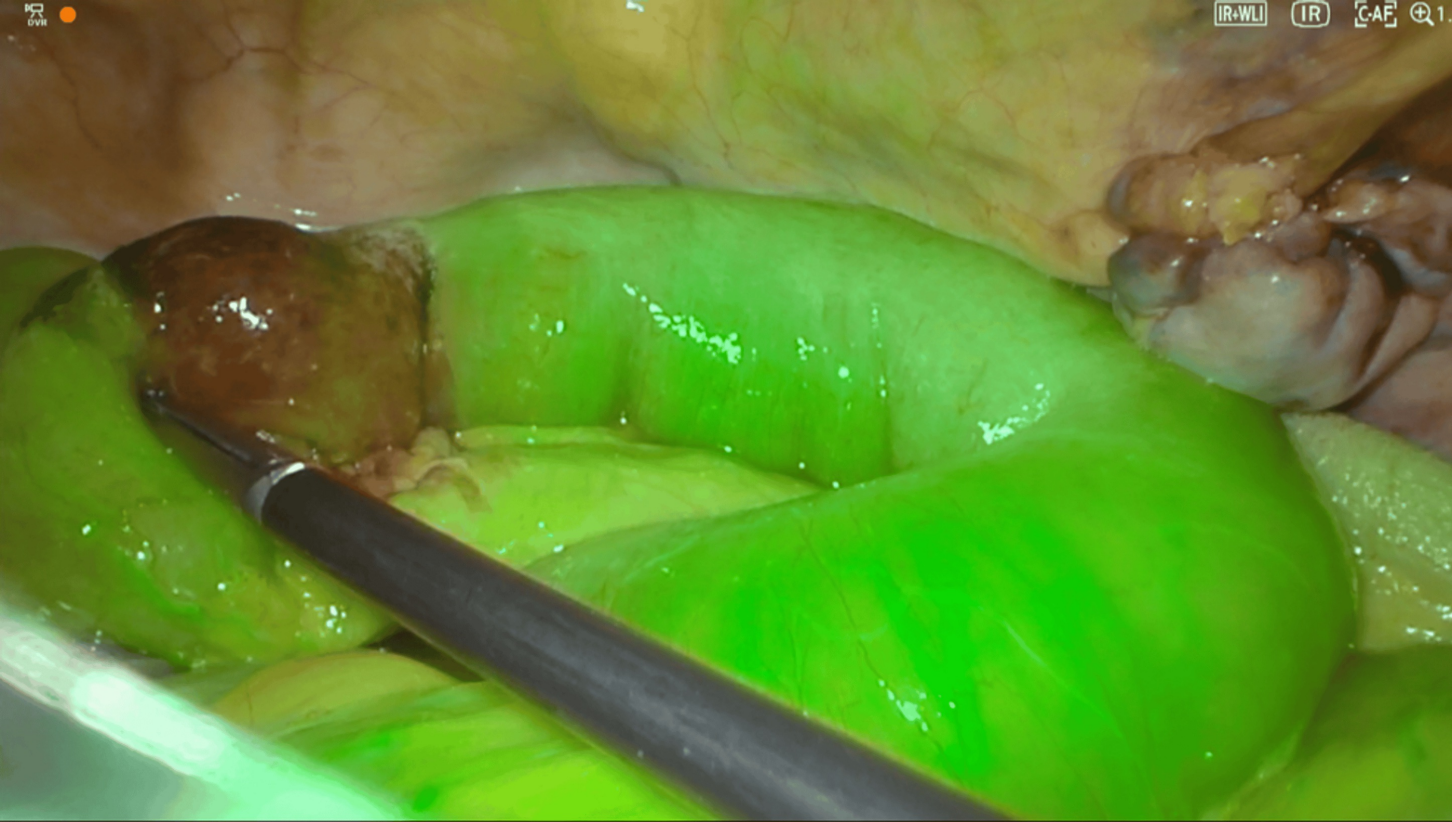
Indocyanine green fluorescence demonstrating bowel perfusion and infarcted area This image was obtained intraoperatively following intravenous administration of indocyanine green at a dose of 0.5 mg/kg (approximately 3 mg), delivered as a bolus followed by a 10 mL saline flush. The lack of green fluorescence in the bowel segment clearly indicates absent perfusion. Based on this finding, an intestinal resection was deemed necessary. Due to operative considerations, the resection and hand-sewn anastomosis were performed through a 2 cm peri-umbilical incision at the site of the optical trocar.

Bowel viability was then assessed using intravenous ICG fluorescence. Perfusion mapping revealed a 3 cm infarcted segment of the ileum (Figure 8). To minimize the risk of mesh contamination and postoperative infection, strict separation between the clean and contaminated fields was maintained. The bowel was exteriorized through a protected mini-umbilical incision only after closure of the preperitoneal space and mesh placement, and it was reintroduced into the peritoneal cavity following completion of the anastomosis. Perioperative antibiotic prophylaxis was administered according to international guidelines for emergency hernia repair involving bowel resection. A limited small-bowel resection was performed, followed by a two-layer, hand-sewn, isoperistaltic anastomosis through a mini-umbilical incision. The postoperative course was uneventful, and the patient was discharged on postoperative day five.

## Discussion

Obturator hernia occurs when intra-abdominal contents protrude through the obturator canal, located in the cranial portion of the obturator foramen and bordered superolaterally by the superior pubic ramus and by musculoaponeurotic structures medially and inferiorly [4]. Because of its deep anatomic location and rarity, accounting for only 0.07%-1% of all hernias, obturator hernia often poses diagnostic and therapeutic challenges [1]. Anatomically and pathophysiologically, the first stage of development involves preperitoneal fat sliding into the obturator canal, forming a so-called “fat plug.” In the second stage, a peritoneal dimple and true sac develop, and in the third stage, peritoneal contents herniate, causing symptoms depending on the structures involved [8]. Predisposing factors include a wider pelvis and triangular obturator canal typical of women, advanced age (which reduces preperitoneal fat and lymphatic tissue), multiparity, and chronic increases in intra-abdominal pressure. For these reasons, it has been called the “little-old-lady’s hernia” [1,4,6].

The most frequent presentation is mechanical bowel obstruction, although about one-third of cases manifest as Richter’s hernia, producing intermittent abdominal pain that subsides when the herniated content spontaneously reduces, complicating diagnosis [5,6]. Physical examination findings are often negative because the hernia lies deep beneath the pectineus and adductor muscles. The pathognomonic Howship-Romberg sign, present in 15%-50% of cases, consists of pain along the medial thigh or hip that worsens with extension, adduction, or internal rotation and improves with flexion [4-6]. Since intestinal obstruction is the most common presentation, preoperative diagnosis is often made via abdominal CT scan, which offers a diagnostic accuracy of approximately 87%. However, even with modern imaging, the rates of strangulated hernia and postoperative morbidity and mortality remain high, as the time from symptom onset to treatment typically ranges from 4.4 to nine days [8], similar to our case, in which five days elapsed before surgery. This delay explains the high rate of intestinal resection (25%-75%) and mortality (15%-50%) reported in the literature [9].

In this context, emergency laparoscopy and intraoperative bowel perfusion assessment using ICG may help reduce unnecessary intestinal resections. Several surgical approaches have been described for obturator hernia repair, including extraperitoneal techniques such as the inguinotomy or totally extraperitoneal (TEP) laparoscopic approach, and intraperitoneal techniques such as the open subumbilical or laparoscopic TAPP approach. In emergency cases, intraperitoneal approaches are preferable as they allow complete assessment of bowel viability and, if necessary, perform an intestinal resection [1]. The hernia defect can be repaired either by simple suture closure for small defects (<1 cm) or with mesh placement for larger ones [1].

The use of prosthetic mesh in emergency inguinal, femoral, and obturator hernia repair-especially in clean-contaminated settings-remains a matter of debate. Reported mesh-related infection rates range from 0.5% to 7% and do not appear to significantly increase in the presence of bowel ischemia requiring resection, while true mesh rejection remains exceedingly rare when adequate preventive measures are adopted [10]. According to the HerniaSurge Group guidelines, the use of macroporous, non-absorbable mesh can be recommended even in clean-contaminated operative fields, although the quality of supporting evidence is still limited [11]. Furthermore, when the surgical field is classified as clean-contaminated and no enteric spillage occurs, current studies indicate that the risk of surgical site infection (SSI) or mesh infection does not increase, whereas prosthetic repair significantly reduces recurrence rates compared with primary suture closure [12,13]. These data support the use of mesh even in emergency settings, provided that contamination is carefully controlled. In our case, no perforation or peritonitis was present, and the mesh was placed before bowel manipulation, minimizing exposure to intestinal content. This strategy aims to reduce the high recurrence risk (up to 30%) associated with primary suture repair.

Despite bowel distension caused by obstruction, laparoscopy offers excellent exposure of the deep obturator foramen and facilitates creation of a wide preperitoneal pocket for mesh placement. In our case, a self-gripping polyester mesh was positioned in the preperitoneal space, and the resection-anastomosis was performed extracorporeally through a small peri-umbilical incision, minimizing operative time (approximately 90 minutes) and peritoneal contamination.

Although the use of laparoscopy in cases of bowel obstruction remains debated, it has been shown to reduce postoperative morbidity, hospital stay, bleeding, and complications [1,6]. Furthermore, laparoscopy allows assessment of the entire myopectineal orifice, enabling detection or repair of concomitant inguinal or femoral hernias. As demonstrated in our case, the laparoscopic approach also permits the use of ICG fluorescence to assess the vitality of the reduced bowel loop and guide intraoperative decision-making.

## Conclusions

Obturator hernia is a rare but potentially life-threatening condition that remains a diagnostic and therapeutic challenge. Our experience suggests that laparoscopic TAPP repair using synthetic mesh can be safely performed even when bowel resection is required, provided that contamination is strictly minimized. This approach may be particularly beneficial in frail patients, reducing recurrence risk without increasing the likelihood of mesh-related complications.

The adjunctive use of ICG fluorescence imaging offers an objective assessment of intestinal perfusion, improving surgical decision-making and helping to avoid unnecessary bowel resections. Incorporating fluorescence-guided laparoscopy in emergency hernia repair may therefore enhance both surgical precision and patient outcomes.
